# Molecular Assessment of Genetic Diversity and Genetic Structure of *Rhanterium epapposum* Oliv. in Scarce Populations in Some Regions of Western Saudi Arabia

**DOI:** 10.3390/plants11121560

**Published:** 2022-06-13

**Authors:** Hassan Mansour, Hameed Alsamadany, Zaki M. Al-Hasawi

**Affiliations:** 1 Department of Biological Sciences, College of Science & Arts, King Abdulaziz University, Rabigh 21911, Saudi Arabia; zalhasawy@kau.edu.sa; 2 Department of Botany, Faculty of Science, Suez Canal University, Ismailia 41522, Egypt; 3 Department of Biological Sciences, Faculty of Science, King Abdulaziz University, Jeddah 21589, Saudi Arabia; halsamadani@kau.edu.sa

**Keywords:** *Rhanterium epapposum*, conservation, populations, genetic diversity, Rabigh

## Abstract

*Rhanterium epapposum* Oliv. is a perennial medicinal shrub growing mainly in desert habitats in the Arabian Peninsula. In western Saudi Arabia, the remaining few populations of this species are exposed to many threats, including overcutting, overgrazing, and recently, increasing human activities. These threats are predicted to be exacerbated by the advancement of aridification caused by climate change. The conservation and recovering of the diminished populations of *R. epapposum* necessitate measurement of their genetic diversity and genetic differentiation. To accomplish this objective, we tested 150 simple sequence repeat (SSR) primer pairs, with which 40 polymorphic loci were identified. These polymorphic loci were used to determine the population genetics of 540 plant accessions sampled from a total of 45 populations of *R. epapposum* located in 8 sites in western Saudi Arabia: Wadi Khurieba, Wadi Al Khamas, Gebel Al Twaal, Al Asaafer, Wadi ALHamda, Wadi Al Nassayeif, Wadi Qaraba, Wadi Kuliayah, and Wadi Dahban. Low levels of genetic diversity were found in all populations (the values of the *PPL* ranged between 52.5 and 15) along with a declined value of *H*_T_ (0.123) and a considerable inbreeding value (*F* = 0.942), which confirmed a noticeable shortage of heterozygotes. High genetic differentiation among the populations and a low value of gene flow are indicative of high isolation among the *R. epapposum* populations, which has caused a severe deficiency in gene migration. The data obtained herein inspire several recommendations for conservation and retrieval of the existing populations, including seed banks, restoration of diminished populations, and monitoring and prevention of cutting and grazing activities at threatened sites. All of these measures are urgently required to avoid imminent extinction.

## 1. Introduction

*Rhanterium epapposum* Oliv. (Asteraceae) is a medicinal and persistent shrub growing in xeric habitats of north-western Africa and the Arabian Peninsula. *R. epapposum* is the only species of its genus found and disseminated throughout Saudi Arabia [1]. Its dried leaves are applied as a spice in food preparation. Local inhabitants collect it for fuel during outdoor picnics when other woody trees are unavailable. In folk medicine, it is a good cure for skin contaminations and gastrointestinal pains. In Sudan and some Afro-Asian regions, it is well-known as an insecticide [2]. It also serves as good fodder for camels and sheep. The species favours sand dunes and hillsides.

*Rhanterium epapposum* populations are limited mainly to valleys, low-level plains, and medium-level mountains in western Saudi Arabia. The apparent smallness of *R. epapposum* populations could be due to the elevated contemporary arid environmental conditions in the region [3]. Human-driven impacts, in addition to cumulative climate change factors [4,5], are expected to cause a further decline in the population sizes of *R. epapposum* and other important plant species in desert habitats such as the Arabian Peninsula.

*Rhanterium epapposum* plants may be subject to low genetic diversity as a result of coincided genetic drift, which is a major reason for depleted fitness and the diminished ability of some populations to adapt to environmental changes [6,7,8]. Therefore, explicating the genetics and structure of the plant populations of *R. epapposum* is necessary to conserve and retrieve this plant species [9], and may contribute to the conservation of other plant species [10].

The main concern for plant species with small population sizes is their ability to perform outcrossing and pursue successful reproduction in extreme arid habitats. For *R. epapposum*, its self-incompatible mating system is considered an extra limitation affecting its persistence [11]. As a member of the family Asteraceae, its floral structure is recognizable by the variable structure of the style papillae, which is categorized as both receptive and non-receptive and is considered to promote outcrossing [12].

Plant populations in xeric habitats with this reproductive system are enduring threats to their survival, including deficiencies in polymorphic genes, genetic drift, and inbreeding [13,14]. Because of co-occurring climate change and over-exploitation of natural resources, many plant populations in the western valleys of the Arabian Peninsula, including *R. epapposum*, are now confronted with the threat of extinction, due to collapses in population size and the subsequent erosion of genetic diversity. For assessing genetic diversity and genetic structure, microsatellite DNA loci are considered to be among the most reliable markers; they offer a precise measure of genetic variation because of their ability to reveal variable repeat regions of the genome. They are referred to as co-dominant genetic markers [15]. Moreover, microsatellite DNA was applied successfully to measure genetic diversity in other rare and wild plant species [10,16,17].

The present study represents an insight into the distribution, genetic diversity, and genetic structure of *R. epapposum* utilizing microsatellite loci. The resulting data on the inbreeding levels within *R. epapposum* populations constitute necessary information for design conservation strategies. The proposed genetic analyses of this species will also support the preservation of its genetic diversity.

## 2. Materials and Methods

### 2.1. Plant Material

**Figure 1:**
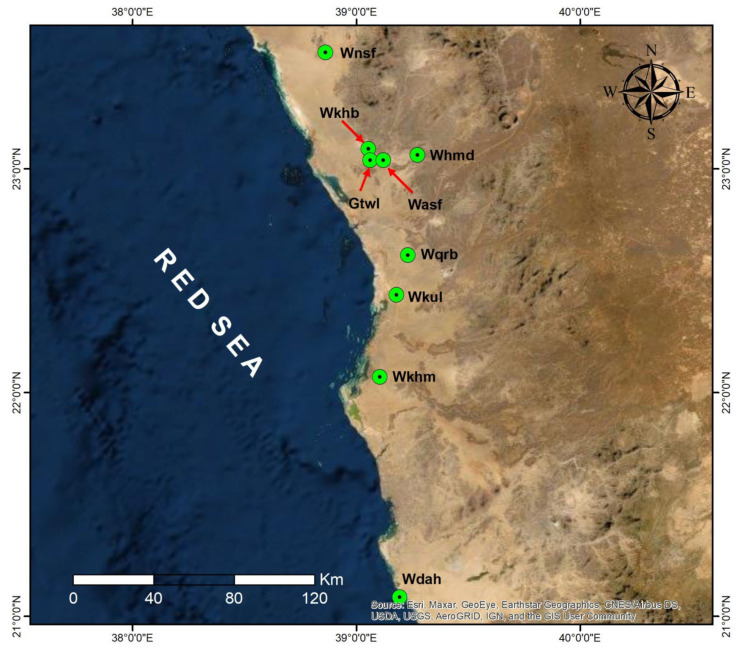
The studied locations of the existing populations of *R. epapposum*.

Forty-five populations of *R. epapposum* were sampled from nine sites in the valleys and mountains in certain western regions of Saudi Arabia (Table 1, Figure 1). Five populations were chosen for sampling from each site. The largest population sampled, with 67 individuals, was the Gtwl 3 population on the Gebel (mountain) Al Twaal site, a mountainous region located north of Rabigh city (Figure 1). The remaining populations contained numbers of individuals ranging between 17 and 66. The lowest number of individuals was 15, recorded for the Wdah 1 population in the Wadi (valley) Dahban site, located to the north of Jeddah city.

From each population, 12 plants were chosen for genotyping analyses using 40 microsatellite loci. After cutting, between one and three leaves were directly immersed in liquid nitrogen in 50 mL labelled falcon tubes and were preserved in a −20 °C freezer until DNA extraction.

### 2.2. Genomic DNA Isolation and PCR Amplification

DNA was isolated from leaf samples collected from a total of 540 plant accessions by a DNeasy Plant Mini Kit (Qiagen, Hombrechtikon, Switzerland). A total of forty-four primers were tested for polymorphisms, using published primers that were successfully designed and tested for other related species in the family Asteraceae [18,19,20]. Forty loci exhibiting polymorphisms were tested for each individual (Table 2). PCR reactions were performed with a master mix of 25 μL containing 2.5 μL of 10× reaction buffer, 1 μL of MgCl_2_ (50 mM), 0.5 μL of a dNTP mix, 0.2 μL of a forward primer (including the M13-tail (10 μM)), 0.5 μL of a reverse primer (10 μM), 0.5 μL of the universal M13 primer (10 µM) labelled with a fluorophore (FAM, NED, VIC, or PET), 0.1 μL of Taq DNA polymerase (Dream Tag, Fermentas; 50 U/μL), 1.0 μL of bovine serum albumin (20 mg/mL), 1.0 μL of 10 ng/µL genomic DNA, and dH2O up to the final volume. All the PCRs were conducted as singleplex assays with a C1000 Thermal Cycler (BioRad, Hercules, CA, USA). The reactions were conducted under the following conditions: initial denaturation at 94 °C for 5 min; 50 cycles at 94 °C for 35 s, 50 cycles at 55 °C for 40 s, and 50 cycles at 72 °C for 50 s; 8 cycles at 94 °C for 30 s, 8 cycles at 53 °C for 45 s, and 8 cycles at 72 °C for 1 min; and a final extension step at 72 °C for 5 min. The fluorescently tagged PCR products were tested in multiplexes on a 3130xl Genetic Analyzer (Applied Biosystems, Foster City, CA, USA) with LIZ500 (Applied Biosystems, Foster City, CA, USA) as a size standard. The Electropherograms of amplified fragments were detected using GeneMapper 4.0 (Applied Biosystems, Foster City, CA, USA), and the lengths of the amplified fragments ranged from 112 to 300 bp in accordance with Arif et al., 2010 [21].

### 2.3. Population Genetic Analysis

The measurement of the parameters of genetic diversity, genetic structure, and inbreeding was conducted using GenAlEx 6.1 [22]. The genetic differentiation among the populations was computed with *R*_ST_, corresponding to *F*_ST_ developed for microsatellite loci [23]. The genetic structure of *R. epapposum* populations was assessed by AMOVA (999 permutations))[24,25]. The number of migrants per generation that performed successful reproduction (*Nm*) was determined by the private allele method [26]. The established heterozygosity (*H*_o_), the expected heterozygosity (*H*_e_) under Hardy–Weinberg equilibrium, and Wright’s fixation index (*F* = 1−*H*_o_/*H*_e_) were evaluated for each locus in each population to test deviations from the Hardy–Weinberg equilibrium, which determines inbreeding. UPGMA dendrogram and principal component analysis (PCA) were implemented using PAST 4.02 [27] based on the following genetic diversity variables: number of alleles Na, number of effective alleles Ne, Shannon information index I, number of private alleles, the expected heterozygosity (*H*_e_), heterozygosity (*H*_o_), and percentage of polymorphic loci (P).

## 3. Results

A total of 40 loci exhibited polymorphisms. The percentage of polymorphic loci (Table 3) was at its highest value (52.5) in the Wkhb 1 population in Khurieba, while the lowest percentage of polymorphic loci (15) was found in the Wdah 2 population in Wadi Dahban. High selfing was suggested by our results for *R. epapposum*, as the average fixation index (*F*) was equal to 0.587, confirming an explicit deficit of heterozygotes (Table 3).

The mean number of alleles per locus (*N_a_*) ranged between 1.75 (Wkhb 1 population) and 1.225 (Wdah 2 population). The means of the number of effective alleles per locus (*N_e_*), Shannon index (*I*), and expected heterozygosity (*H_e_*) varied from 1.492, 0.369, and 0.229 in the Wkhb1 population, respectively, to 1.108, 0.085, and 0. 0.047 in the Wdah 2 population, respectively (Table 3). The average total heterozygosity (*H*_T_) for all the loci and populations was 0.123.

**Figure 2:**
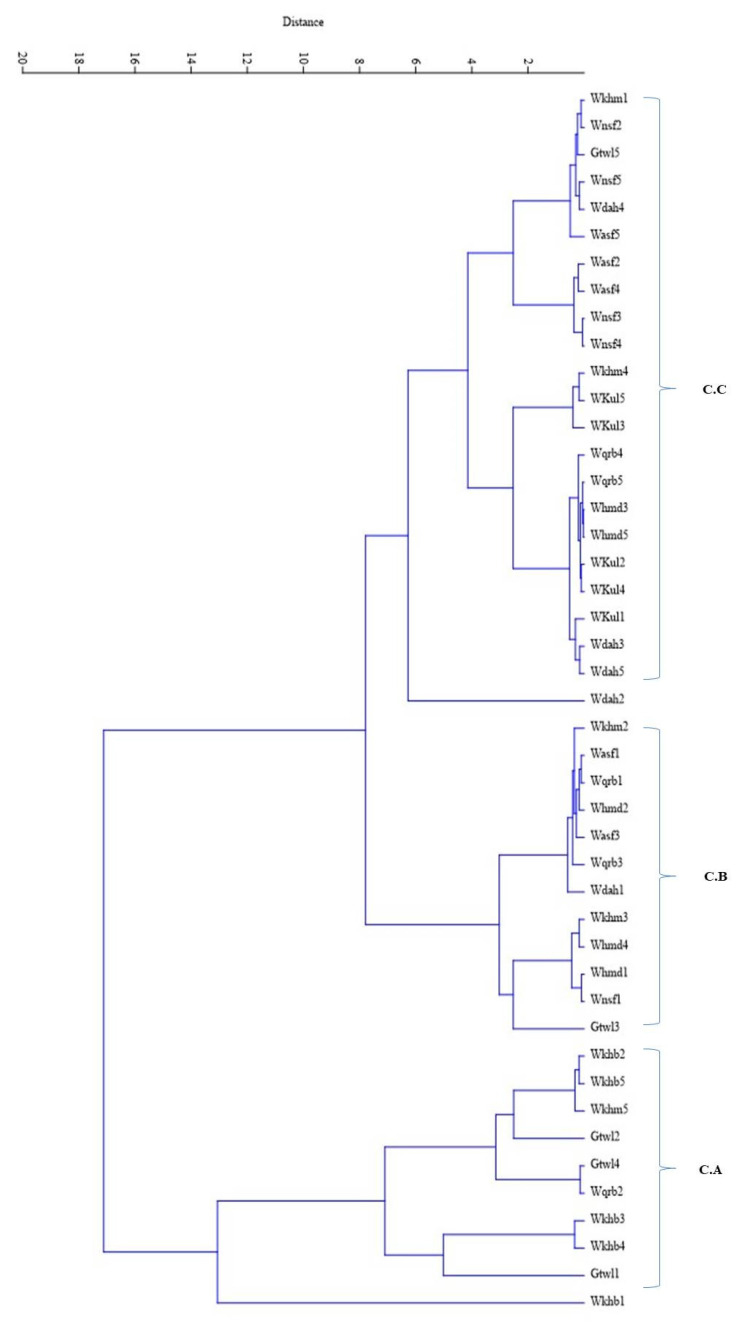
UPGMA dendrogram for the 20 populations of *R. epapposum*.

The cluster analysis is shown on a UPGMA dendrogram (Figure 2) subdivided into three main clusters. The first cluster, C.A., included all populations from Wadi Khurieba, as well as three populations from Gebel Al Twaal (Gtwl 1, Gtwl 2, and Gtwl 4), which exhibited the highest means of genetic variables; the remaining populations were distributed in the second cluster, C.B. and the third cluster, C.C. The second cluster contained three populations from the Wadi ALHamda site (Whmd 1, Whmd 2, and Whmd4) and two populations from the Wadi Al Khamas site (Wkhm 2 and Wkhm 3). The last cluster, C.C., had four populations from Wadi Dahban (Wdah 2, Wdah 3, Wdah 5, and Wdah 4), all the sampled populations of the Wadi Kuliayah site, three populations from the Wadi Al Nassayeif site (Wnsf 2, Wnsf 3, and Wnsf 4), and three populations from the Wadi Al Asaafer site (Wasf 2, Wasf 4, and Wasf 5), these sites showing the lowest values of genetic diversity variables and being geographically isolated from Wadi Khurieba and Gebel Al Twaal.

**Figure 3:**
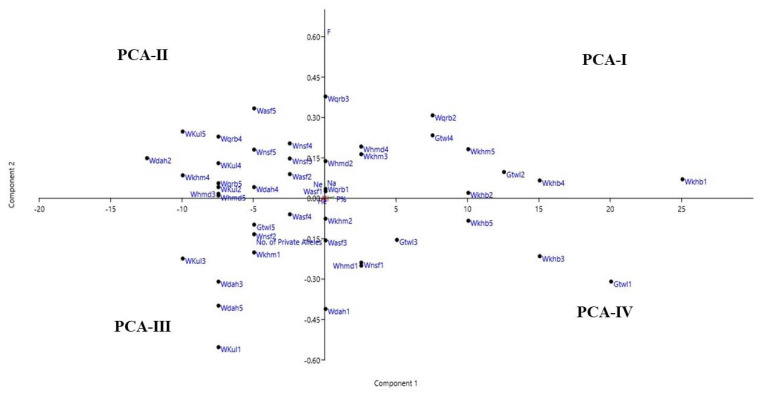
Principle component analysis (PCA) implemented for the studied populations of *R. epapposum*.

The PCA results (Figure 3) showed three out of seven principal components were significant (Eigen value >1) and contributed 99.9% of the total variation. The three significant components were *N*_a,_ accounting for 73.4, *F*, accounting for 6.9, and *P*, accounting for 4.9. The PCA explains the variance among the studied sites, and it categorized the *R. epapposum* populations into four groups. Group PCA-I included the populations from Wadi Khurieba and Gebel Al Twaal that maintain favourable conditions of high altitude mountain and water reserves and that assist *R. epapposum* to maintain slightly higher genetic diversity parameters and variance than other populations in the remaining sites. This group includes: three populations of Wadi Khurieba, three populations of Gebel Al Twaal, and three populations of Wadi Qaraba; group PCA-II included three populations from Wadi Al Asaafer, two populations from Wadi Al Nassayeif, three populations from Wadi Kuliayah, and two populations from Wadi ALHamda; group PCA-III aggregates populations from sites in Wadi Dahban, Wadi Kuliayah, and Wadi Al Asaafer, where unfavourable environmental conditions pose negative effects on genetic diversity parameters, and it included three populations from Wadi Dahban, two populations from Wadi Kuliayah, two populations from Wadi Al Asaafer, and two populations from Wadi Al Khamas. The remaining populations from Gebel Al Twaal, Wadi Khurieba, Wadi Al Nassayeif, and Wadi Al Khamas were included in PCA-IV, which aggregated in this group due to declining genetic diversity parameters as a consequence of extra stress from overgrazing and human overutilization in these sites.

This finding confirmed the UPGMA cluster analysis, e.g., resemblance existed between PCA-I and first cluster C.A. The AMOVA revealed considerable genetic differentiation among the studied *R. epapposum* populations (*R*_ST_ = 0.894). The highest genetic differentiation occurred between the populations (89%, *P* = 0.001), whereas the lowest value (1%, *P* = 0.010) was detected among individuals within the populations.

## 4. Discussion

In the current analysis of the molecular genetics of *R. epapposum*, all the genetic diversity parameters revealed a modest to severe decline in values among all the remaining populations studied, in accordance with other studies on the same species growing in arid habitats of Kuwait [11]. Moreover, many studies have been performed on related plant species of Asteraceae, with different species showing a considerable reduction in gene diversity and encountering similar harsh environmental conditions; [16,17,28,29,30,31].

The main reasons behind the modest to severe decline in genetic diversity of these populations may be due to their low population size. As a result of the limited population size, the existing *R. epapposum* populations are vulnerable to severe genetic drift and inbreeding, which worsens the problem of the polymorphic allele loss due to the ambient environmental circumstances [32,33,34].

The pattern of genetic diversity distribution among the studied locations showed substantial variability, with a relatively moderate polymorphism determined in the populations of Wadi Khurieba, Gebel Al Twaal, Wadi Qaraba, and Wadi Al Khamas, which could be explained by the relative abundance of water reserves in these regions. Wadi Khurieba is situated to the south of Bany Ayoub Mountain, where many water aggregations are available from frequent inundations of rain in this site. The small gorges in Gebel Al Twaal (700–900 m a.s.l) permit the growth of plant populations, as they have access to more water. Wadi Qaraba and Wadi Al Khamas are low-level valleys with more water reserves, permitting the growth of many plant populations. Al-Gharaibeh et al. 2017 [35] mentioned the same relationship between the maintenance of genetic diversity in the populations of other plant species and water accessibility and altitude in the desert.

The PCA confirmed the relationship between the proportion of genetic diversity and the availability of water resources. The populations with higher polymorphisms were grouped in the PCA-I group, which included populations from Wadi Khurieba, Gebel Al Twaal, and Wadi Qaraba.

The high inter-population genetic differentiation values obtained by AMOVA analysis is inferred as relating to the high isolation among the studied sites. The main factors leading to this isolation can be summarized in terms of local human activities, including overcutting and severe overgrazing by camels and sheep, which increases during spring time, as these herds are transported by their owners from drier regions during this season [36]. Moreover, water resources are subjected to overuse due to current industrial developments [3,37].

Greater declines in existing population size and further isolation are expected with co-occurring higher temperatures and drier conditions, which are evident in the decreasing frequency of rain in these regions [4,5]. Moreover, the prolonged effect of high temperature could impose extra strain on the reproductive potential of *R. epapposum* flowers, as it has a negative effect on pollination and will thus likely increase the possibility of selfing [38].

Despite the fact that the morphology and size of the *R. epapposum* fruit of the achene type, with its membranous cover, facilitate its dispersal over long distances, a noticeably low level of gene migration among existing populations in the current study suggests that the extensive anthropogenic and climatic causes of isolation have contributed to elevating the value of genetic differentiation among the studied populations. This is confirmed by the population divergence, as shown in the UPGMA dendrogram [16,39].

The values revealed in the gene flow assessment fell short of the values necessary for stopping the increase in genetic drift [40]. The joint effect of genetic drift and gene flow could worsen the future decline in gene diversity in the remaining populations of *R. epapposum*.

## 5. Conclusions and Recommendations

Our current research represents a first assessment of the genetic diversity and genetic structure of *R. epapposum* in habitats of western Saudi Arabia, and was conducted in order to help manage conservation actions to protect this valuable medicinal species. The species faces imminent extinction due to a severe decline in gene polymorphism coupled with high inter-population genetic differentiation and considerable inbreeding.

The long-term plan for the conservation of *R. epapposum* should be primarily based on decreasing the degradation and deterioration of its current habitats. Many actions can be taken in this regard, including the following. Firstly, wire-fenced enclosures around the populations severely affected by low genetic polymorphism can be established [41], as identified in our current study, e.g., Wadi Al Khamas, Al Asaafer, Wadi ALHamda, Wadi Al Nassayeif, Wadi Kuliayah, and Wadi Dahban. These enclosures are highly recommended to prevent camel and sheep herds from grazing on these sites, and should be monitored regularly to measure vegetation parameters and observe any further changes occurring in the existing protected populations. Secondly, management plans should be devised to reduce water consumption and to promote the reuse of wastewater and the efficient use and storage of water from sudden rains. These plans should be widely broadcasted to the inhabitants of the western regions through media and educational institutes. Their aim should be to ensure the efficient utilization of underground water.

Thirdly, the evident decline in genetic diversity and the high genetic differentiation in these populations support the idea that they could be restored by collection and preservation of *R. epapposum* seeds from all the remaining populations [42].

The collected seeds would primarily be involved in *R. epapposum* recuperation programmes, in which the seeds would be planted in nurseries and the well-developed seedlings would then be reintroduced into highly threatened populations. The seedlings would be reintroduced into habitats resembling those of their parent populations in order to reduce potential ramifications, including further inbreeding and severe decreases in gene flow. Some of the collected healthy seeds should be protected using appropriate seed-maintenance protocols in special test banks; these would be valuable for future efforts to conserve *R. epapposum* in its original habitats.

## Figures and Tables

**Table 1:** Sites and population information: acronyms used to refer for populations, coordinates of sites, and population size of the twenty known populations of *R. epapposum* in a region of western Saudi Arabia.

Population Site	Population Acronym	Longitude (E)	Latitude (N)	Total No. of Individuals
Wadi Khurieba	Wkhb 1	39°3′12.6252″	23°5′21.2892″	66
	Wkhb 2			63
	Wkhb 3			58
	Wkhb 4			60
	Wkhb 5			52
Wadi Al Khamas	Wkhm 1	39°6′18″	22°48′10.8″	42
	Wkhm 2			36
	Wkhm 3			38
	Wkhm 4			32
	Wkhm 5			33
Gebel Al Twaal	Gtwl 1	39°3′39.6″	23°2′20.4″	57
	Gtwl 2			49
	Gtwl 3			67
	Gtwl 4			59
	Gtwl 5			53
Wadi Al Asaafer	Wasf 1	23°2′17.016″	39°7′15.6″	49
	Wasf 2			42
	Wasf 3			45
	Wasf 4			52
	Wasf 5			44
Wadi Qaraba	Wqrb 1	22°36′50.4″	39°13′48″	63
	Wqrb 2			55
	Wqrb 3			57
	Wqrb 4			44
	Wqrb 5			49
Wadi ALHamda	Whmd 1	23°3′45.72″	39°16′22.8″	22
	Whmd 2			39
	Whmd 3			37
	Whmd 4			28
	Whmd 5			31
Wadi Al Nassayeif	Wnsf 1	23°31′15.6″	38°51′43.2″	26
	Wnsf 2			28
	Wnsf 3			29
	Wnsf 4			19
	Wnsf 5			22
Wadi Kuliayah	WKul 1	22°26′13.2″	39°10′47.28″	27
	WKul 2			25
	WKul 3			33
	WKul 4			22
	WKul 5			19
Wadi Dahban	Wdah 1	21°57′3.6″	39°11′37.32″	15
	Wdah 2			17
	Wdah 3			21
	Wdah 4			19
	Wdah 5			25

**Table 2:** Status of SSR primers used.

Marker		Ta(°C)	SSR Motif	Size of PCR Products	References
Mm 01	F: CGAAATTGCCCTCTTCTTCC	60	(CA)_15_(CT)_8_	188–211	[18]
	R: TCCTCCAGCTTCCTCTTCAA				
Mm 02	F: GCGGGAACGGATAGTTACAA	56	(AC)_8_(TC)_2_	187–203	
	R: TCGTGTTCCTCTCGATGTCA				
Mm 05	F: GCGGGAACGGATAGTTACAA R: TCGTGTTCCTCTCGATGTCA	62	(CT)_6_AAAAA(CA)_9_	183–186	
Mm 07	F: TGGTTCTTATTTGAGCCCAATC R: TCGGTTATCGCAATAATAAATGG	56	(CATA)_3_(CA)_6_(CATA)_44_	143–284	
Mm 12	F: TGTTTGGAGACTTTGGTTGAGA	60	(CG)_5_(CA)_6_	156–161	
	R:TTTGCATAGTTAGTGAAAACTCACA				
Mm 15	F: TCATGGTTGCCTGTAAACGA	62	(TA)_3_(TG)_9_(TA)_3_	214–223	
	R: TGAAACTGTGCTATGATGAAACG				
Mm 18	F: TCACCCAAACATAAAAGCTTGA	63	(GT)_10_AAC(GT)_5_	200–249	
	R: AAATCACCATCAAACTCATCCA				
Mm 19	F: CGGCCACTTCTTTATTCAGC	62	(GT)_10_	229–241	
	R: CCATGCACACACACAAGGTT				
Mm 20	F: TCATTTCAGCCCAAATCACA	62	(CA)_7_CC(CA)_5_	162–165	
	R:CATTTCTCCCCTCTATATGTATATGTC				
Mm 21	F: GCGATGGTTTTAGGGGTTTT	60	(GT)_6_TT(GT)_2_	161–164	
	R: GACCCTACAACAAGGGACGA				
Mm 27	F: CTTGATTGCACCAGCAACAG	60	(TG)_8_	208–222	
	R: CCACATGCATCAACCCATAA				
Mm 31	F: AAAGGTGGTGCTTGTGTAGTTG	62	(TG)_7_	207–216	
	R: TTGGGTCACGTTTGATTTGA				
MmESP03	F: TGGACATCATTTTCCTCTACCA	60	(AG)_6_	233	[19]
	R: ATGTTCCAATGGGCTGTCTC				
MmESP06	F: AGTTTTTCGCCTCTGCACAC R: CATCGTCTCCACCTTTCACA	60	(AC)_12_	239	
MmESP09	F: TTTGGCCAGGTCTCAAATTC R: CCAACCCAAGGATGAGATTG	60	(AC)_11_	102	
MmESP11	F: AACTCTCCGGTGACAACCAC	60	(CCA)_7_	125	
	R: TTAGACCGCTTGCCTTTGTT				
MmESP12	F: CCAAAGTCTGTGGTGTGCAG R: AGATTGTAAGCCTGGCGATG	60	(CCA)_6_	258	
MmESP13	F: AGGGTTTGATTTGTCCCACA R: GCTGTTGAAGTGCGAAATGA	60	(CAC)_8_	224	
MmESP14	F: CACTTCAATGGCTTCCACCT R: CTTACGATTTTGCGGGATTG	60	(TCA)_6_	153	
MmESP19	F: GCCGGTAACTCTCTCAACCA R: GGAGACAAGAGACGCCGTAG	60	(CTT)_12_	268	
MmESP21	F: CCGTGACGAGAAACAACTCA R: ATTTACCGACGACGGAGATG	60	(GAT)_6_	208	
MmESP23	F: CTGTGCCTTGTTTTGCTTCA R: TGAGCTTTTGGGGAAGAAGA	60	(ATG)_6_	182	
MmESP25	F: TGTCACGCAAAACACACTCA R: ACGAACTTAAACGGCACGTC	60	(CTCAT)_5_	269	
MmESP28	F: AGCTCCCTCCGACTCATTTT R: TCAGAGCTTCACATGGTCGT	60	(AC)_9_(TC)_6_	267	
MmESP30	F: ATTCACGACGACTTCCCTCA R: CCCAGAACCCTAAACACCAA	60	(TC)_10_	207	
MmESP35	F: AAAATGGGCAACTGTCAAGC R: CACGAAGACGTTGATTGGTG	60	(ACC)6	244	
MmESP37	F: TTGTAGTGCTTTCCGGTGTG R:GAGGAGAGTAAACCGGTGGAG	60	(CAC)_5_	191	
MmESP40	F: TTGCCATTTTGTTGTCGTTG R: TCCAAGGGGCATATCCATAC	60	(TAC)_6_	189	
Cl3	F: TGATTCCCCATCATCGAATAATA	58	(TAA)_6_	166–202	[20]
	R: TCCTATCTTCTCTCCGTTTCCAT				
Cl12	F: AATCACTTCACCATGAGGATGAC	58	(CCA)_6_	207–216	
	R: ACAGGAAGGGTTCAAAATCCTA				
Cl23	F: AATAGGCTTTTCACCTTTTCCTC	59	(TAT)_7_	159–162	
	R: TTGATTGGTAGTTGAAAACTTGC				
Cl28	F:CACACACTATAACCACAAACTCGAT	60	(AG)_10_	220–244	
	R: CTCCACCACACCATAAGATGAA				
Cl42	F: TTCTTTCACAATCGTTCATTTCA	60	(TTA)_6_	227–230	
	R: GATCACCTGCTAAAATCACGAAC				
Cl52	F: TGGTTCTAGTCTTAACACGTGGG	60	(AAT)_6_	214–220	
	R: ACAACTCCCCTGTATCCAAAAAT				
Cl76	F: GCTCCAGTTTCACCTAGAAAGAA	60	(GAT)_6_	212–245	
	R:TCACACAATATTTCTAAAACTACATCAA				
Cl84	F: AACCGTTGTTTGATTACACTCGT	60	(GAT)_6_	140–155	
	R: AGAAGGTTTCTTGAACTTGGAGG				
Cl92	F: TGGATCACCGTTTTCTTCTTAAA	60	(AGC)_6_	103–112	
	R: ACCACCTATTCCAACATCTTCCT				
Cl95	F: TCAAAGTACACATCACTACCCCA	60	(AT)_10_	160–172	
	R: AATAAGAAGAAGAAATGGCGGG				
Un6	F: TAATGGGCTCAGTAACACCTCTG	60	(AGA)_6_	116–122	
	R: ATCACGATCGCAAACAGAAAC				
Un23	F: TCTTGGAACATGGAGATTCAACT	58	(TCA)_6_	130–139	
	R: GAAGAGTGCACGAGTTCAGTAGG				

**Table 3:** The mean values of the of genetic diversity variables across the studied populations of *R. epapposum*: *N*_a_ (no. of different alleles), *N*_e_ (no. of effective alleles), *I* (Shannon’s information index), no. private alleles (no. of alleles unique to a single population), *H_e_* (expected heterozygosity), P (the percentage of polymorphic loci), and *F* (fixation index) in the twenty studied populations of *R. epapposum*.

Population	*N*_a_	*N*_e_	*I*	No. of Private Alleles	*H*_e_	*P*%	*F*
Wkhb 1	1.750	1.492	0.369	0.250	0.229	52.50	0.455
Wkhb 2	1.575	1.353	0.264	0.200	0.162	37.50	0.555
Wkhb 3	1.675	1.391	0.304	0.375	0.184	42.50	0.297
Wkhb 4	1.725	1.404	0.291	0.125	0.170	42.50	0.520
Wkhb 5	1.675	1.429	0.284	0.275	0.166	37.50	0.450
Wkhm 1	1.300	1.180	0.144	0.050	0.088	22.50	0.464
Wkhm 2	1.350	1.187	0.167	0.050	0.105	27.50	0.544
Wkhm 3	1.625	1.377	0.261	0.025	0.151	30.00	0.719
Wkhm 4	1.325	1.177	0.130	0.000	0.075	17.50	0.792
Wkhm 5	1.625	1.277	0.241	0.000	0.143	37.50	0.675
Gtwl 1	1.725	1.430	0.330	0.175	0.201	47.50	0.094
Gtwl 2	1.675	1.452	0.303	0.250	0.180	40.00	0.607
Gtwl 3	1.425	1.231	0.177	0.225	0.105	32.50	0.452
Gtwl 4	1.600	1.378	0.267	0.100	0.161	35.00	0.769
Gtwl 5	1.400	1.265	0.183	0.200	0.111	22.50	0.592
Wasf 1	1.600	1.422	0.251	0.025	0.144	27.50	0.597
Wasf 2	1.550	1.405	0.233	0.075	0.132	25.00	0.705
Wasf 3	1.625	1.449	0.269	0.050	0.154	27.50	0.408
Wasf 4	1.650	1.475	0.257	0.100	0.137	25.00	0.540
Wasf 5	1.600	1.425	0.228	0.025	0.123	22.50	0.962
Wqrb 1	1.550	1.388	0.243	0.100	0.141	27.50	0.633
Wqrb 2	1.675	1.459	0.293	0.025	0.170	35.00	0.812
Wqrb 3	1.550	1.355	0.231	0.000	0.132	27.50	0.968
Wqrb 4	1.425	1.289	0.180	0.025	0.102	20.00	0.905
Wqrb 5	1.400	1.276	0.183	0.025	0.107	20.00	0.728
Whmd 1	1.525	1.343	0.235	0.100	0.139	30.00	0.321
Whmd 2	1.500	1.327	0.217	0.075	0.125	27.50	0.745
Whmd 3	1.375	1.266	0.169	0.025	0.100	20.00	0.691
Whmd 4	1.475	1.324	0.225	0.050	0.136	30.00	0.775
Whmd 5	1.375	1.256	0.167	0.050	0.097	20.00	0.692
Wnsf 1	1.575	1.368	0.247	0.025	0.144	30.00	0.306
Wnsf 2	1.375	1.214	0.159	0.000	0.092	22.50	0.511
Wnsf 3	1.375	1.257	0.180	0.000	0.111	25.00	0.777
Wnsf 4	1.375	1.250	0.177	0.000	0.109	25.00	0.836
Wnsf 5	1.350	1.204	0.153	0.025	0.090	22.50	0.848
WKul 1	1.325	1.205	0.142	0.075	0.084	20.00	0.122
WKul 2	1.325	1.198	0.141	0.000	0.083	20.00	0.722
WKul 3	1.300	1.189	0.129	0.075	0.076	17.50	0.491
WKul 4	1.325	1.188	0.136	0.025	0.079	20.00	0.822
WKul 5	1.300	1.198	0.135	0.025	0.080	17.50	0.968
Wdah 1	1.475	1.273	0.200	0.450	0.116	27.50	0.278
Wdah 2	1.225	1.108	0.085	0.250	0.047	15.00	0.962
Wdah 3	1.300	1.144	0.121	0.200	0.070	20.00	0.416
Wdah 4	1.350	1.180	0.139	0.150	0.082	22.50	0.738
Wdah 5	1.350	1.228	0.157	0.275	0.092	20.00	0.329
Overall mean	1.481	1.304	0.209	0.103	0.123	27.44	0.587

## Data Availability

Not applicable.

## References

[1] Collenette S. (1999). Wildflowers of Saudi Arabia.

[2] Shama I.Y., Shama I.Y.A., Adam S.E.I. (2012). Comparative toxicity of Trichodesma africanum and Rhanterium epapposum aerial parts aqueous and methanolic extracts on Wistar rats. J. Pharmacol. Toxicol..

[3] Tarawneh Q.Y., Chowdhury S. (2018). Trends of Climate Change in Saudi Arabia: Implications on Water Resources. Climate.

[4] Issar A.S., Zereini F., Hötzl H. (2008). The impact of global warming on the water resources of the Middle East: Past, present and future. Climate Changes and Water Resources in the Middle East and North Africa.

[5] Soultan A., Wikelski M., Safi K. (2019). Risk of biodiversity collapse under climate change in the Afro-Arabian region. Sci. Rep..

[6] Luijten S.H., Dierick A., Gerard J., Oostermeijer B., Raijmann L.E.J., Den Nijs H.C.M. (2000). Population size, genetic variation, and reproductive success in a rapidly declining, self incompatible perennial (*Arnica montana*) in the Netherlands. Conserv. Biol..

[7] Hansson B., Westerberg L. (2002). On the correlation between heterozygosity and fitness in natural populations. Mol. Ecol..

[8] Bastiaan S., Hamish G.S. (2013). Effects of genetic drift and gene flow on the selective maintenance of genetic variation. Genetics.

[9] Upendra J.M., Rao S.R., Dagla R. (2017). Genetic diversity analysis of Salvadora persica: An evergreen halo-xeric species of semi-arid and sub-humid regions of Rajasthan, India. Ecol. Genet. Genom..

[10] Hatmaker E.A., Staton M.E., Dattilo A.J., Hadziabdic D., Rinehart T.A., Schilling E.E., Trigiano R.N., Wadl P.A. (2018). Population Structure and Genetic Diversity within the endangered species *Pityopsis ruthii* (Asteraceae). Front. Plant Sci..

[11] Al Salameen F., Habibi N., Al Amad S., Kumar V., Dashti J., Talebi L., Al Doaij B. (2020). Genetic diversity analysis of *Rhanterium eppaposum* Oliv. by ISSRs reveals a weak population structure. Curr. Plant Biol..

[12] Katinas L., Hernández M.P., Arambarri A.M., Funk V.A. (2016). The origin of the bifurcating style in Asteraceae (Compositae). Ann. Bot..

[13] Keller L.F., Waller D.M. (2002). Inbreeding effects in wild populations. Trends Ecol. And. Evol..

[14] Vilas C., San Miguel E., Amaro R., García C. (2005). Relative contribution of inbreeding depression and eroded adaptive diversity to extinction risk in small populations of shore campion. Conserv. Biol..

[15] Kim K.S., Sappington T.W., Kantartzi S. (2013). Microsatellite Data Analysis for Population Genetics. Microsatellites: Methods in Molecular Biology (Methods and Protocols).

[16] Mansour H., Sliwinska E. (2017). Genetic Diversity and Inbreeding Level of Cotoneaster orbicularis Schltdl. in The Sinai Mountains Revealed by Microsatellite Markers and Flow Cytometry. Egypt. J. Bot..

[17] Mansour H., Alsamadany H., Al-Hasawi Z.M. (2020). Genetic diversity and genetic structure of Salvadora persica L., rare plant species in Rabigh province, Saudi Arabia: Implications for conservation. J. Taibah Univ. Sci..

[18] Hong L., Niu H., Shen H., Ye W., Cao H. (2008). Development and characterization of microsatellite markers for the invasive weed *Mikania micrantha* (Asteraceae). Mol. Ecol. Resour..

[19] Yan Y., Huang Y., Fang X., Lu L., Zhou R., Ge X., Shi S. (2011). Development and characterization of EST-SSR markers in the invasive weed *Mikania micrantha* (Asteraceae). Am. J. Bot..

[20] Luo L., Zhang P., Ou X., Geng Y. (2016). Development of EST-SSR markers for the invasive plant *Tithonia diversifolia* (Asteraceae). Appl. Plant Sci..

[21] Arif I.A., Khan H.A., Shobrak M., Al Homaidan A.A., Al Sadoon M., Al Farhan A.H., Bahkali A.H. (2010). Interpretation of electrophoretograms of seven microsatellite loci to determine the genetic diversity of the Arabian Oryx. Genet. Mol. Res..

[22] Peakall R., Smouse P.E. (2012). GenAlEx v.6.5: Genetic analysis in Excel. Population genetic software for teaching and research. Bioinformatics.

[23] Slatkin M. (1995). A measure of population subdivision based on microsatellite allele frequencies. Genetics.

[24] Excoffier L., Smouse P.E., Quattro J.M. (1992). Analysis of molecular variance inferred from metric distances among DNA haplotypes: Application to human mitochondrial DNA restriction data. Genetics.

[25] Michalakis Y., Excoffier L. (1996). A generic estimation of population subdivision using distances between alleles with special reference for microsatellite loci. Genetics.

[26] Barton N.H., Slatkin M. (1986). A Quasi-equilibrium theory of the distribution of rare alleles in a subdivided population. Heredity.

[27] Hammer Ø., Harper D.A.T., Ryan P.D. (2001). PAST: Paleontological statistics software package for education and data analysis. Palaeontol. Electron..

[28] Wang L., Liu J., Jian S., Zhang W., Wang Q., Zhao X., Liu N., Zhong Y. (2006). Genetic diversity and population structure in Elephantopus scaber (Asteraceae) from South China as revealed by ISSR markers. Plant Biosyst..

[29] Abd El-Twab M.H., Zahran F.A. (2010). RAPD, ISSR and RFLP analysis of phylogenetic relationships among congeneric species (Anthemideae, Asteraceae) in Egypt. Int. J. Bot..

[30] Crowford D.J., Ruiz E., Stuessy T.F., Tepe E., Aqeveque P., Gonzalez F., Jensen R.J., Anderson G.J., Bernardello G., Baeza C.M. (2011). Allozyme diversity in endemic flowering plant species of the Juan Fernandez Archipelago, Chile: Ecological and historical factors with implications for conservation. Am. J. Bot..

[31] Hirai M., Kubo N., Ohsako T., Utsumi T. (2012). Genetic diversity in the endangered coastal violet *Viola grayi* Franchet et Savatier (Violaceae) and its genetic relationship to the species in subsection *Rostratae*. Conserv. Genet..

[32] Blomqvist D., Pauliny A., Larsson M., Flodin L.A. (2010). Trapped in the extinction vortex? Strong genetic effects in a declining vertebrate population. BMC Evol. Biol..

[33] Jacquemyn H., Roldán-Ruiz I., Honnay O. (2010). Evidence for demographic bottlenecks and limited gene flow leading to low genetic diversity in a rare thistle. Conserv. Genet..

[34] Smyser T.J., Duchamp J.E., Johnson S.A., Larkin J.L., Rhodes O.E. (2012). Consequences of metapopulation collapse: Comparison of genetic attributes between two Allegheny woodrat metapopulations. Conserv. Genet..

[35] Al-Gharaibeh M.M., Hamasha H.R., Rosche C., Lachmuth S., Wesche K., Hensen I. (2017). Environmental gradients shape the genetic structure of two medicinal *Salvia* species in Jordan. Plant Biol..

[36] Al-Rowaily S.L., El-Bana M.I., Al-Bakre D.A., Assaeed A.M., Hegazy A.K., Ali M.B. (2015). Effects of open grazing and livestock exclusion on floristic composition and diversity in natural ecosystem of Western Saudi Arabia. Saudi J. Biol. Sci..

[37] Harter T., Davis H., Mathews M., Meyer R. (2002). Shallow ground water quality on dairy farms with irrigated forage crops. J. Contam. Hydrol..

[38] Root T.L., Price J.T., Hall K.R., Schneider S.H., Rosenzweig C., Pounds J.A. (2003). Fingerprints of global warming on wild animals and plants. Nature.

[39] Jimenez A., Mansour H., Keller B., Conti E. (2014). Low genetic diversity and a high level of inbreeding in the Sinai primrose (*Primula boveana*), a species on the brink of extinction. Plant Syst. Evol..

[40] Spieth P.T. (1974). Gene flow and genetic differentiation. Genetics.

[41] Koyama A., Uchida K., Ozeki M., Iwasaki T., Nakahama N., Suka T. (2021). Conservation of endangered and rare plants requires strategies additional to deer-proof fencing for conservation of sub-alpine plant diversity. Appl. Veg. Sci..

[42] Holsinger K.E., Gottlieb L.D., Falk D.A., Holsinger K.E. (1991). Conservation of rare and endangered plants: Principles and prospects. Genetics and Conservation of Rare Plants.

